# Increased levels of CGRP in peripheral blood in women with chronic migraine: A reliable biological marker

**DOI:** 10.1186/1129-2377-14-S1-P104

**Published:** 2013-02-21

**Authors:** E Cernuda-Morollon, D Larrosa, G Moris, C Ramon, J Pascual

**Affiliations:** 1 University of Oviedo, Spain; 2 University Hospital Central de Asturias, Spain

## Introduction

The biology of chronic migraine (CM) as a true entity or as a consequence of analgesic overuse is controversial. There are no available biological markers for CM, while CGRP, a marker of trigemino-vascular activation, has been shown to be increased during acute migraine attacks in episodic migraine [1].

## Objectives

To determine CGRP levels in peripheral blood in a series of patients with CM as compared with matched subjects without a headache history.

## Patients and methods

This series comprises 61 women meeting CM diagnostic criteria (IHC-II 2006 revised) with a mean age of 44 years (range 16-63) and 19 women (41 years, 22-55) without any headache history. CGRP levels were determined in blood samples obtained from right cubital vein between 9-12 am with an ELISA kit from USCN following manufacturer's instructions. Acute medication had not been taken the day before.

## Results

CGRP levels were increased in women with CM (77.94 ng/ml, range 27.69-157.72) as compared to controls (40.39 ng/ml, range 20.08-70.75) (+93%; p<10-8).

## Conclusions

CGRP levels are clearly increased in patients with CM, which is compatible with a permanent activation of trigemino-vascular system in this entity. CGRP determination may constitute the first reliable biological marker for CM.
